# RV work efficiency is greatly reduced in patients with pulmonary arterial hypertension as evidenced by 4D flow cardiac MRI

**DOI:** 10.1186/1532-429X-16-S1-P235

**Published:** 2014-01-16

**Authors:** Qiao Han, Walter R Witschey, Jefferey Arkles, Alex J Barker, Yuchi Han

**Affiliations:** 1 Cardiology, University of Pennsylvania, Philadelphia, Pennsylvania, USA; 2 Surgery, University of Pennsylvania, Philadelphia, Pennsylvania, USA; 3 Northwestern University, Chicago, Illinois, USA

## Background

The dynamic assessment of intracardiac flow remained challenging until the recent development of time-resolved 3D phase-contrast CMR (PC-CMR), which allows for assessment of multi-dimensional whole heart and great vessel flow. Previous studies have used this technique in the ventricles to quantify flow type of the ventricle and the kinetic energy of each flow component. In the great vessels, parameters such as total flow, peak systolic velocity, and wall shear stress have excellent scan-rescan, inter-observer, and intra-observer reproducibility. In disease conditions, pathological vortices have been reported both in the ventricles and great vessels. We propose to use time-resolved 3D PC-CMR to study right ventricular (RV) work in patients with pulmonary arterial hypertension (PAH) as compared to normal subjects.

## Methods

Five healthy subjects and eight patients with PAH underwent cardiac MRI exams, in which 2D cine short axis slices, 2D and 4D flow were acquired. The 4D flow data were acquired during free breathing using an ECG-gated 3D cine PC-MRI sequence on a 1.5T Siemens Avanto scanner (Germany) with the following parameters: Venc = 125 cm/s, flip angle = 8°, voxel size = 2.50 × 2.50 × 2.50 mm^3^, and bandwidth = 400 Hz, slab thickness about 60-70 mm covering the complete RV and at least the main PA. Acquisition time is 17 to 25 minutes. Following the flow acquisition, the flow data were reconstructed into 13-21 time frames. Total kinetic energy through tricuspid and pulmonic valves were calculated with diastolic and systolic durations respectively. RV workload and RV efficiency were defined and calculated as the following: RV workload=(KE_out_−KE_in_)/KE_out _, and RV efficiency = 1-RV workload, where KE_in _and KE_out _correspond to the kinetic energy coming in and out of the two RV valves.

## Results

**Figure 1:**
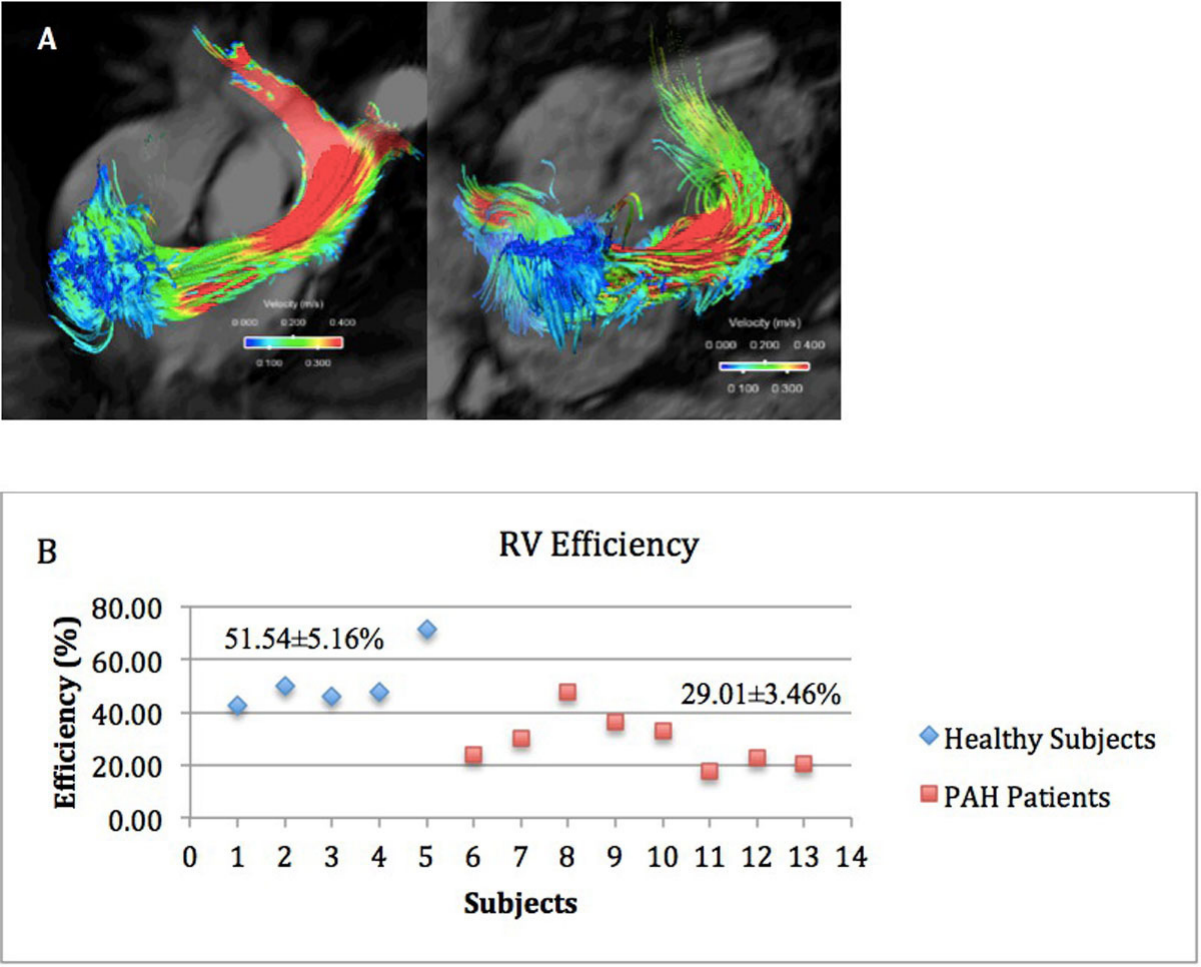
**(A) RV inflow and outflow of a control subject (left) and a PAH patient (right)**. (B) RV Efficiency for 13 study subjects. Mean RV efficiency of healthy subjects is significantly greater than that of PAH patients, with p-value = 0.007.

Six female and two male PAH patients were included in the study (mean age = 44.9 years). Left ventricular ejection fraction (EF) ranged between 53 to 72%. The RVEF ranged from 40 to 54%. RV mass and RV end_diastolic volume were increased in 70% of patients. The blood flow through the RV into the pulmonary artery can be visualized as the pathlines formed by the velocity vectors obtained from the scans (Figure 1A). Healthy subjects showed significantly higher RV efficiency than PAH patients (51.54 ± 5.16% and 29.01 ± 3.46%, p = 0.007) (Figure 1B). Moreover, RV efficiency does not significantly correlate with any of the RV structural parameters.

## Conclusions

In conclusion, this study has quantified the kinetic energy input and output through the RV over the cardiac cycle, and estimated the lower bound of work done by the RV as the difference between the kinetic energy coming in from the tricuspid valve and out of the pulmonic valve. RV efficiency, independent from other existing RV parameters, appears to provide a potential new metric to assess RV function. This initial approach opens the door to more precise quantifications.

## Funding

This research was funded through FOCUS Junior Faculty Investigator Award for Research in Women's Cardiovascular Health supported by the Edna G. Kynett Memorial Foundation.

